# Complex Pulmonary Arteriovenous Fistula Presented With Ventricular Tachycardia and Type 2 Acute Myocardial Infarction

**DOI:** 10.7759/cureus.61347

**Published:** 2024-05-30

**Authors:** Ming Ren Ma, Liang Shi, Fei Wang, Xiaoqing Cai, Ling Ma

**Affiliations:** 1 Cardiology, 940th Hospital, Joint Logistics Support Force, Chinese People's Liberation Army (PLA), Lanzhou, CHN

**Keywords:** cardiac surgery, myocardial infarction, ventricular tachycardia, chest distress, pulmonary arteriovenous fistula

## Abstract

Pulmonary arteriovenous fistula (PAVF) is a rare congenital vascular malformation primarily manifested as dyspnea, migraine, ischemic stroke, hemoptysis, and nervous system complications. However, in our case, an 18-year-old male patient with PAVF presented with sudden onset of ventricular tachycardia and type 2 acute myocardial infarction as initial symptoms. A diagnosis was achieved through pulmonary artery computer tomography angiography (CTA) and three-dimensional (3D) computed tomography (CT) reconstruction, revealing a complex and giant PAVF. Following multidisciplinary team (MDT) consultation, the patient underwent thoracoscopic surgery and experienced a successful recovery during follow-up.

## Introduction

Pulmonary arteriovenous fistula (PAVF) is a rare disorder of pulmonary vascular development [1]. This is characterized by varying degrees of right-to-left shunts, with an incidence of approximately two to three per 100,000 cases [2]. The pathogenesis of PAVF is primarily attributed to the incomplete development of capillaries between the pulmonary artery branches and pulmonary vein plexus during the embryonic stage, resulting in the direct communication between pulmonary arteries and veins. Consequently, PAVF facilitates the direct return of unoxidized venous blood to the pulmonary veins and left heart, leading to a right-to-left shunt and subsequent hypoxia. Over time, this shunt induces significant pathophysiological alterations in the patient’s body and pulmonary circulation, ultimately contributing to a poor prognosis when left untreated [3]. The present case report presents a case of a young male patient diagnosed with PAVF, who initially presented with ventricular tachycardia and type-2 acute myocardial infarction.

## Case presentation

**Figure 1:**
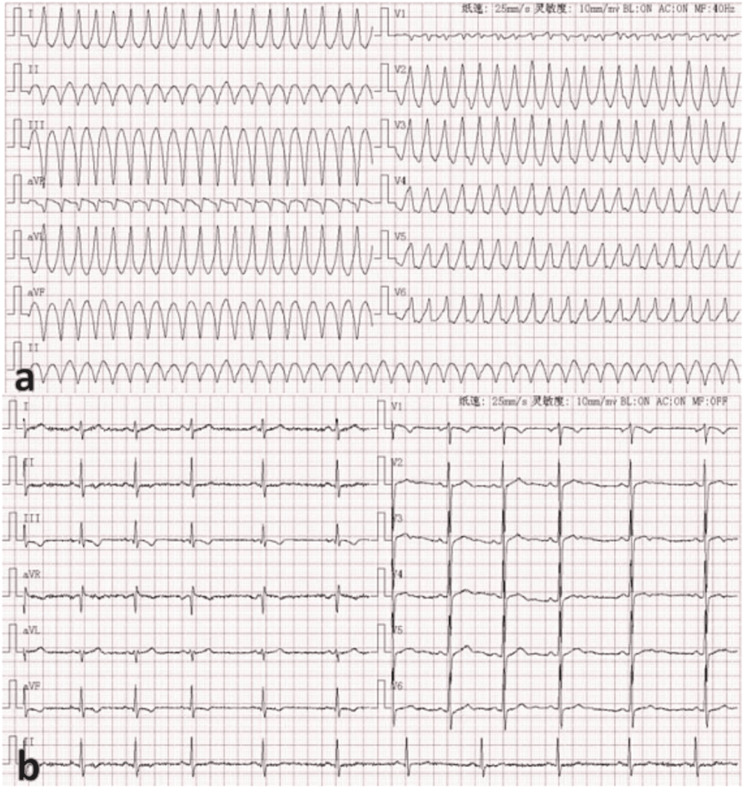
(a) The emergency ECG shows the ventricular tachycardia. (b) The sinus rhythm recovery after emergency cardioversion.

An 18-year-old male patient was admitted to the Emergency Department of our hospital after a sudden episode of syncope. The electrocardiogram (ECG) results revealed ventricular tachycardia (Figure 1a). After receiving urgent cardioversion, the patient’s consciousness recovered within a minute, with the sinus rhythm evident on the ECG (Figure 1b). The patient complained of intermittent chest pain and chest tightness for six months. Furthermore, the patient had no medical history of hypertension, diabetes, coronary heart disease, hepatitis, infection, or fever. However, the patient was diagnosed with pulmonary artery hypertension half a year ago and was treated with sildenafil citrate and furosemide. The physical examination revealed cyanosis of the lips and nail beds, with an augmented heart sound in P2. However, no murmurs were detected.

The laboratory examination results, as presented in Table 1, indicated a hemoglobin level of 211 g/L with a hematocrit of 62.4%. Additionally, the arterial blood gas (ABG) analysis yielded a pH of 7.37, oxygen partial pressure of 43 mmHg, oxygen saturation of 81%, a PaO2/FiO2 (P/F) oxygenation index of 203 mmHg, and a lactic acid level of 2.6 mmol/L. Furthermore, the myocardial zymogram revealed significantly elevated levels of creatine kinase (CK) (443 IU/L) and creatine kinase isoenzymes (CK-MB) (68 IU/L), while the cardiac troponin I (cTnI) level was measured at 0.023 ug/L.

**Table 1:** Preoperative, postoperative, and follow-up laboratory test results. ABG, Arterial blood gas; PaO2, Partial pressure of oxygen; FiO2, Fraction of inspired oxygen; CK, Creatine kinase; CK-MB, Creatine kinase-myocardial band; cTnl, Cardiac troponin I.

	Preoperative	Postoperative	Follow-up visit	Reference value
Hemoglobin (g/L)	211	184	164	131~172
Hematocrit (%)	62.4	53.9	46	42~48
ABG (PH)	7.37	7.4	7.41	7.35~7.45
Oxygen partial pressure (mmHg)	43	71	79	80~100
Oxygen saturation (%)	81	95	97	95~98
PaO2/FiO2 oxygenation index (mmHg)	203	214	375	400~500
Lactic acid (mmol/L)	2.6	2.7	1.4	1.0~1.4
CK (IU/L)	443	63	48	50~310
CK-MB (IU/L)	68	20	12	0~24
cTnI (ug/L)	0.023	0.013	0.015	0~0.026

**Figure 2:**
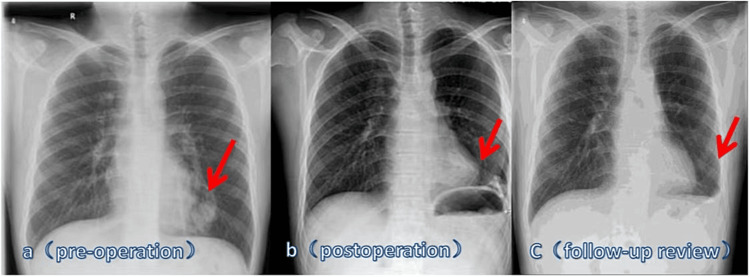
(a) Before the operation, a nodular density with increased shadow was observed in the upper right lung field, accompanied by a lumpy dense shadow adjacent to the left lower lung heart shadow, with an indistinct edge. A strip-shaped vascular shadow is visible above it. (b) After the resection of the left lower lobe and thoracic drainage, pneumothorax occurred on both the left and right sides, with lung tissue compression of approximately 30%. (c) After resection of the left lower lobe and thoracic drainage, the left pneumothorax persisted at a similar level as before.

The imaging examination revealed several significant findings. In digital radiography (DR), a nodular density enhancement shadow was observed in the upper field of the right lung, alongside a lumpy dense shadow with a size of approximately 44 × 33 mm, observed next to the heart shadow of the left lower lung. The marginal part was unclear, and a strip-shaped blood vessel shadow was observed above it (Figure 2a).

**Figure 3:**
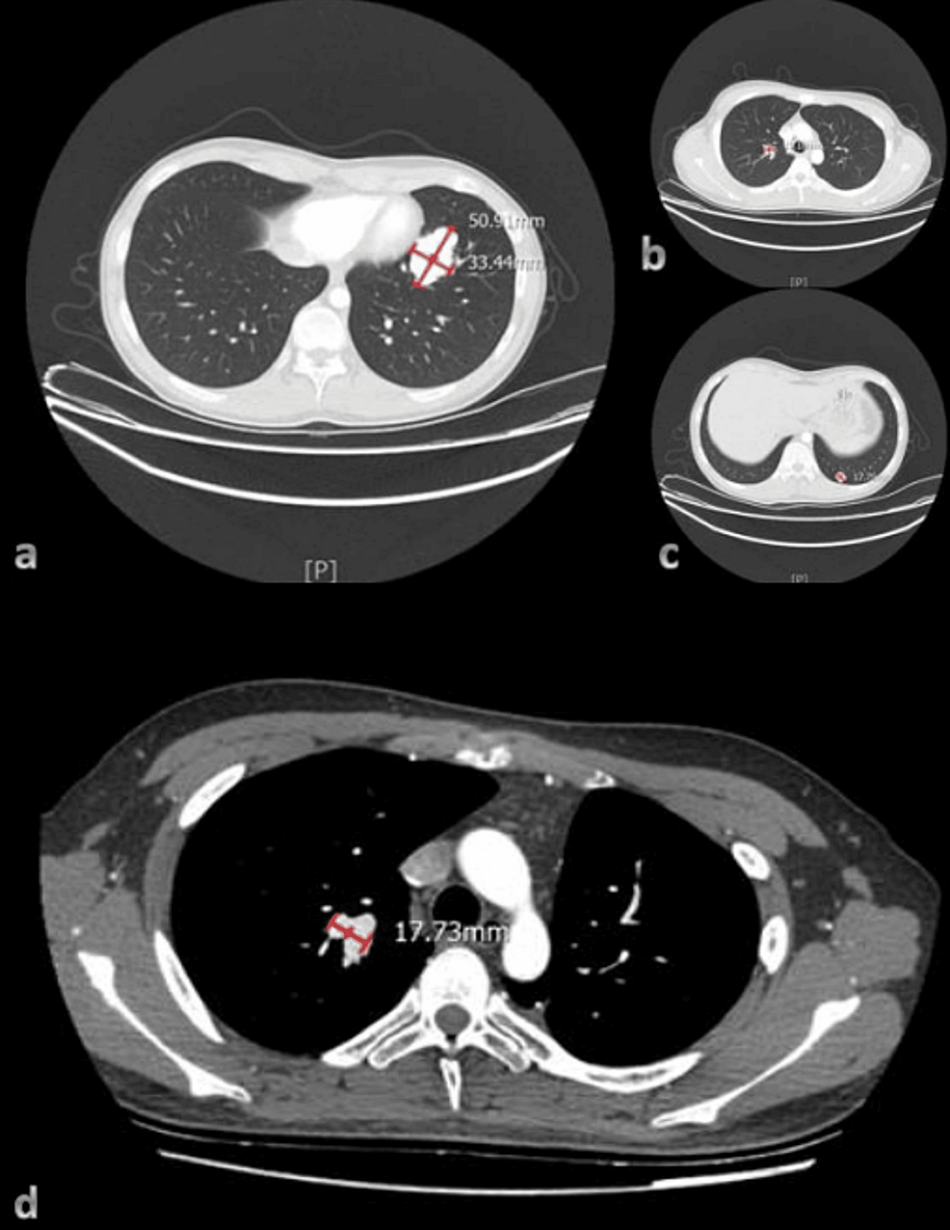
Arteriovenous fistulas were observed in the right upper lobe and left lower lobe of the pulmonary artery computer tomography angiography (CTA), with the following measurements: (a) 50.91 × 33.44 mm, (b) 17.75 × 9.66 mm, and (c) 19.10 × 8.36 mm. At six months after the operation, the arteriovenous fistula in the right upper lobe of the pulmonary artery CTA had a measurement of (d) 17.73 × 9.83 mm. (c) After resection of the left lower lobe and thoracic drainage, the left pneumothorax persisted at a similar level as before.

Computed tomography (CT) confirmed the presence of a thick vascular shadow in both the right upper lobe and left lower lobe. Additionally, pulmonary artery computer tomography angiography (CTA) identified arteriovenous fistulas in the upper right lobe and lower left lobe, measuring 50.91 × 33.44 mm (Figure 3a), 17.75 × 9.66 mm (Figure 3b), and 19.10 × 8.36 mm (Figure 3c) respectively.

**Figure 4:**
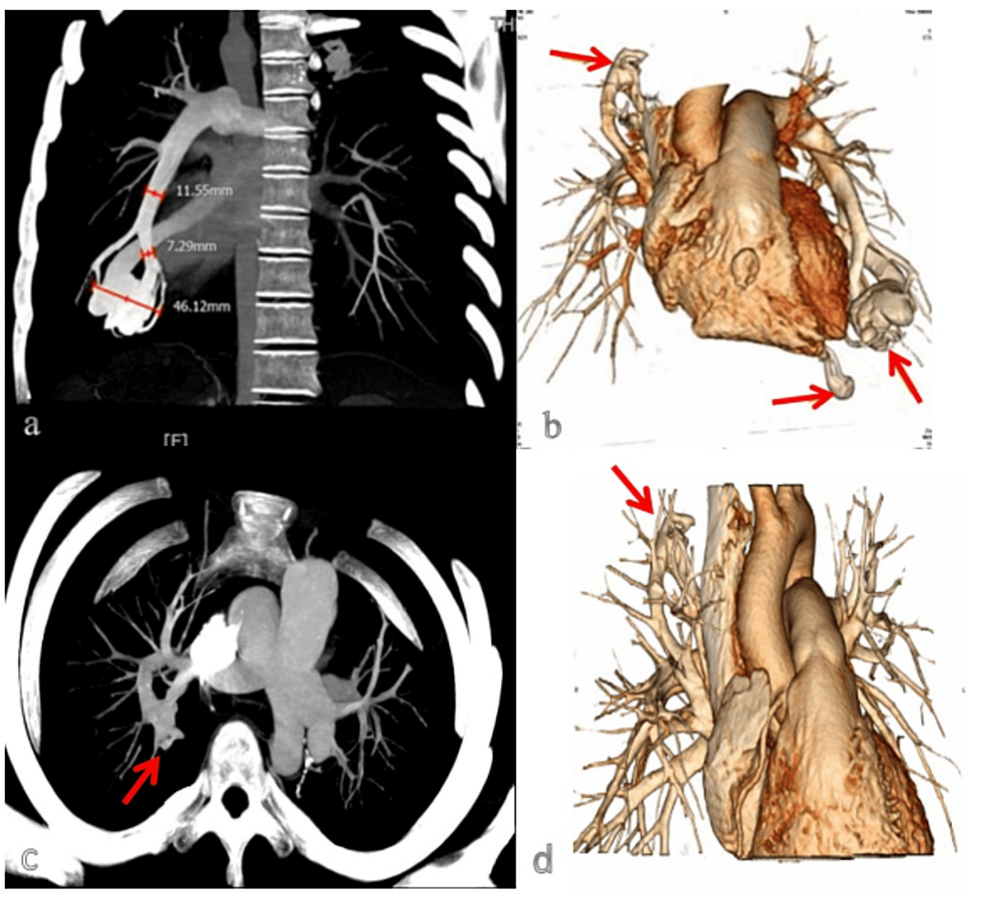
(a) The pulmonary arteriovenous fistula drainage artery diameter was approximately 11.55 mm. (b) The pulmonary artery 3D-CT before the operation. The pulmonary angiography (c) and pulmonary artery 3D-CT (d) at six months after the operation.

Further examination via three-dimensional computed tomography (3D-CT) revealed the drainage artery of the larger arteriovenous fistula to have a diameter of approximately 11.55 mm, with the fistula itself reaching a maximum diameter of 46.12 mm (Figures 4a, 4b).

**Figure 5:**
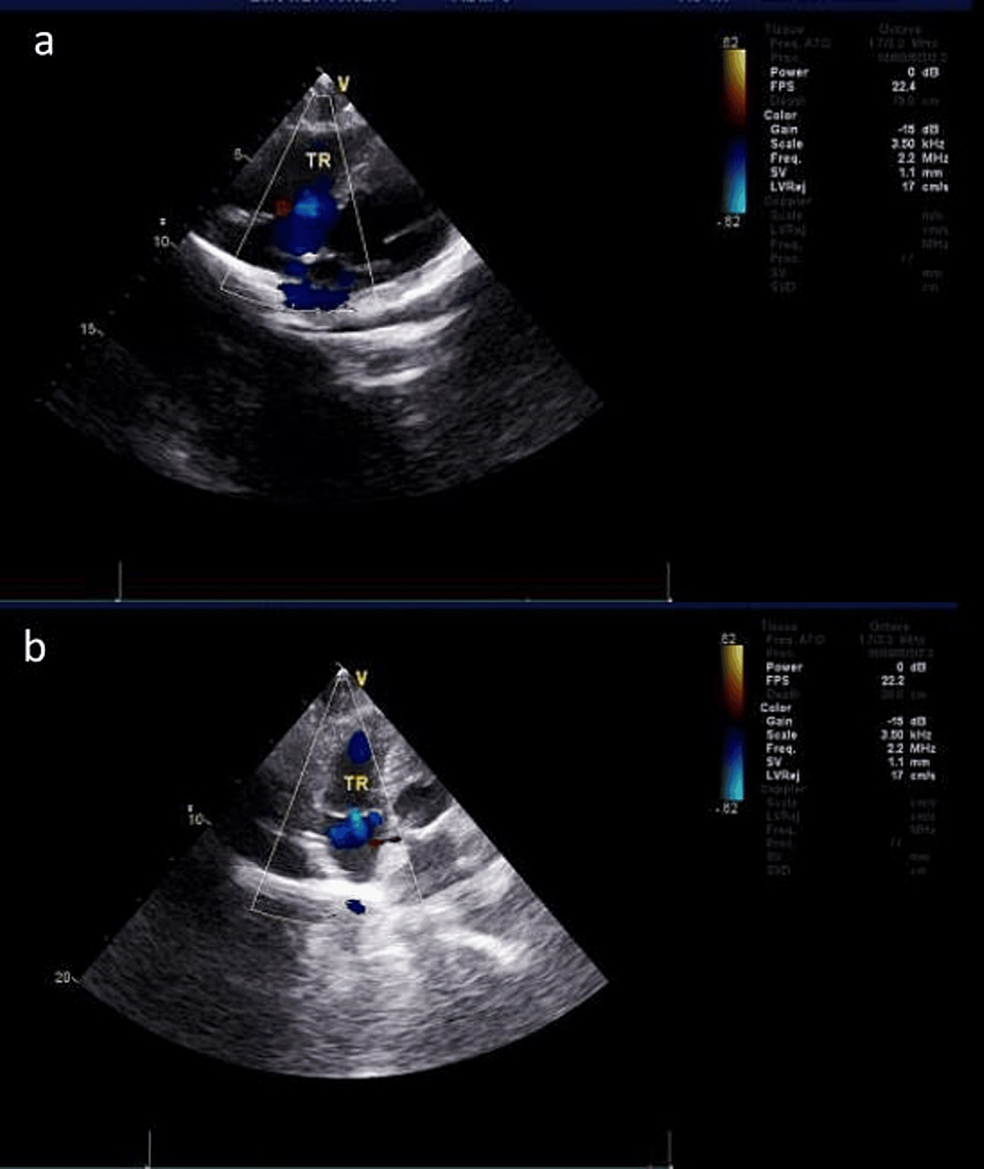
(a) Preoperative ultrasonic cardiogram (UCG): The heart size was within normal limits, with a left ventricular ejection fraction (LVEF) value of 66%. The tricuspid regurgitation velocity was 188 cm/s, the bundle length was 2.3 cm, the area was 1.8 cm2, and the volume was 1 ml. The pulmonary artery systolic pressure was 48 mmHg. (b) The UCG was followed up at six months, post-surgery. The heart size remained normal, with a slightly decreased LVEF value of 64%. No abnormalities were noted in the color of blood flow. The pulmonary artery systolic pressure decreased to 15 mmHg.

Echocardiography results indicated a normal heart size, left ventricular ejection fraction (LVEF) of 66%, tricuspid regurgitation (TR) velocity of 288 cm/s, jet length of 2.3 cm, area of 1.8 cm², and volume of 1 ml in TR, with a calculated pulmonary artery systolic pressure of 48 mmHg (Figure 5a). Other examinations, including color Doppler ultrasound of the blood vessels of both lower limbs, color Doppler ultrasound of the abdomen, and head magnetic resonance imaging, revealed no obvious abnormalities.

The six-minute walk experiment was 350 meters, obviously less than the reference value of 450 meters, indicating a moderately impaired distance. Thus, the diagnosis of complex PAVF was established after considering the multiple-modality imaging results. After the multi-disciplinary team consultation, it was determined that the risk of interventional closure operation was too high to perform. Since transcatheter closure might lead to massive pulmonary embolism and aggravate pulmonary hypertension, it was important to carefully consider the risks and benefits before proceeding with the procedure. Thereafter, a thoracotomy was performed to remove the giant pulmonary arteriovenous fistula, which manifested as a number of brown soft vesicles that reached up to 6 × 4 cm.

After the operation, the patient experienced a notable recovery, with the resolution of chest pain and chest tightness. Furthermore, there were improvements noted in the laboratory results as presented in Table 1. The complete blood count indicated a hemoglobin level of 184 g/L and a hematocrit of 53.9%. ABG analysis showed a pH of 7.4, oxygen partial pressure of 71 mmHg, oxygen saturation of 95%, a P/F oxygenation index of 214 mmHg, and a lactic acid level of 2.7 mmol/L. Additionally, myocardial zymogram results displayed significantly decreased levels of CK (63 IU/L) and CK-MB (20 IU/L), while the cTnI level was measured at 0.013 ug/L. The imaging examinations revealed several findings. The DR examination revealed left pneumothorax post left lower lobe resection combined with thoracic drainage, with lung tissue compression by approximately 30% (Figure 2b). Subsequent to the left lower lobe resection and thoracic drainage, the left pneumothorax persisted at a similar level (Figure 2c).

**Figure 6:**
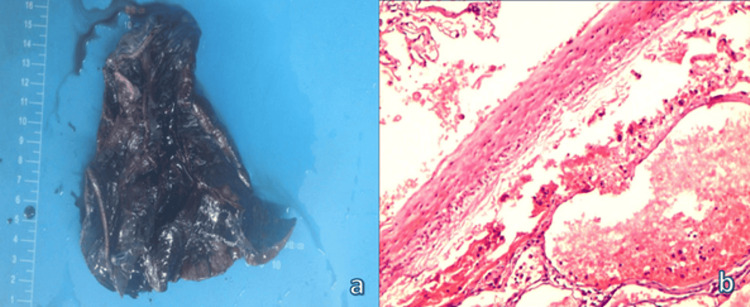
(a) The pathological examination indicates the presence of a lesion in the lower lobe of the left lung, which was 13.0 × 12.0 × 5.5 cm in size. (b) The hematoxylin and eosin staining at 200× magnification revealed the presence of a pulmonary arteriovenous fistula within the lung lobe.

Additionally, the pathological examination of the lung lobectomy specimen (13.0 × 12.0 × 5.5 cm) revealed multilocular cystic dark red nodules that contained bloody fluid, with a blister (0.8 × 0.7 × 0.7 cm) on the surface (Figure 6a). The microscopic analysis revealed numerous thick-walled blood vessels in the lung tissue, which is consistent with vascular malformation (Figure 6b). Furthermore, the echocardiographic evaluation revealed a pulmonary artery systolic pressure of 15 mmHg, leading to the significant alleviation of both hypoxia and pulmonary hypertension. Upon discharge, the patient remained stable and discontinued the medication.

The patient was followed up for six months. The laboratory examination results (Table 1) revealed several findings. The hemoglobin level was measured at 164 g/L, accompanied by a hematocrit of 46%. The ABG analysis indicated a pH of 7.41, an oxygen partial pressure of 79 mmHg, oxygen saturation at 97%, a P/F oxygenation index of 375 mmHg, and a lactic acid level of 1.4 mmol/L. The myocardial zymogram results revealed CK levels at 48 IU/L and CK-MB at 12 IU/L. Lastly, the cTnI level was 0.015 ug/L. Overall, these findings suggest relief from hypoxia. The imaging examinations revealed that the left pneumothorax was reabsorbed. The pulmonary artery CTA revealed residual PAVF in the upper right lung lobe, with minimal deformities noted in the remaining pulmonary artery CTA examination, and a small amount of effusion in the left thoracic cavity (Figure 3d). The heart size remained normal, with an LVEF value of 64%. The color of blood flow was within normal parameters, and the pulmonary artery systolic pressure was 15 mmHg (Figure 5b). The pulmonary angiography and 3D-CT revealed a favorable prognosis, with no evidence of recurrence (Figures 4c, 4d). The six-minute walk test reached 560 meters, indicating a normal range of functionality. During follow-up, the patient exhibited a high level of compliance and expressed satisfaction with the efficacy of the diagnosis and treatment, resulting in a gradual reduction of symptoms.

## Discussion

PAVF was initially proposed by Churton in 1897, and confirmed by Smith’s application of cardiovascular angiography. Its advancements have been observed in its diagnosis and treatment methods over time. Furthermore, this is closely associated with hereditary hemorrhagic telangiectasia (HHT) and is recognized as an important complication of HHT [4,5]. Although rare, PAVF can result from trauma, surgery, liver cirrhosis, or infections [6,7]. Pathologically, PAVF can be categorized into three types: simple, complex, and diffuse. The simple type is more common in clinical settings.

The clinical manifestations of PAVF vary depending on the size of the fistula shunt. Patients are initially asymptomatic, and may later experience chest tightness, dyspnea, cyanosis, clubbing, and exertional dyspnea, due to the decrease in blood oxygen saturation. Complications, such as cerebral infarction, cerebral apoplexy, and peripheral abscess, can also arise. Non-specific symptoms, such as back pain and syncope, may less frequently occur [8].

In the present case, a young patient with complex PAVF presented with cyanosis in the lips and nail beds, intermittent chest pain, and discomfort, suggestive of myocardial ischemia. The chronic right-to-left shunting and pulmonary arterial hypertension, compounded by long-term furosemide use, led to ventricular tachycardia, and ultimately, type-2 acute myocardial infarction. The immediate intervention upon admission, which included cardioversion and potassium supplementation, successfully terminated the ventricular tachycardia.

The diagnostic criteria for PAVF include chest DR, pulmonary artery CTA, and pulmonary artery 3D-CT. Although pulmonary angiography remains the gold standard [9,10], this carries a risk of vessel injury. Pulmonary artery CTA serves as a viable alternative for diagnosis and severity evaluation, especially in complex cases. The present patient’s comprehensive imaging data aided the treatment decision-making.

The therapeutic goals for PAVF focus on reducing or eliminating the shunt. Interventional therapy [11] is preferred due to its minimally invasive nature, and rapid patient recovery. However, for complex cases, such as the present case that had large fistulas, surgery offers thorough treatment, and a lower recurrence rate [12,13]. Furthermore, in the present case, the thoracotomy for left lobe resection was proven to be effective.

Although genetic screening for HHT-related gene mutations, including endoglin (ENG), activin receptor-like kinase 1 (ACVRL1), and drosophila mothers against decapentaplegic protein family member 4 (SMAD4) [14], is essential, this was unfortunately not conducted for the present case. Despite the surgical intervention, small remaining pulmonary arteriovenous orifices pose a risk of recurrence, which should be monitored by ECG and echocardiography during follow-up.

PAVF requires long-term follow-ups due to its chronic, progressive nature, and associated complications [15,16]. Detailed follow-up data would aid in the evaluation of surgical effects, refinement of treatment strategies, and understanding of the disease pathogenesis. The present case highlights the safety and efficacy of surgical resection in improving the patient’s quality of life.

## Conclusions

Pulmonary arteriovenous fistula (PAVF) is a rare yet clinically significant condition. This study presents a case of complex PAVF accompanied by ventricular tachycardia and type 2 acute myocardial infarction, which was successfully managed through surgical resection. After the operation, The patient recovered quickly, was discharged safely, and showed a higher quality of life in the follow-up for six months. Continued monitoring and follow-up of the patient were conducted based on his ECG and echocardiography.

While transcatheter intervention is typically the preferred treatment strategy for PAVF, surgical resection remains a viable option for complicated cases with multiple arteriovenous fistulas. The choice of treatment should be individualized based on the specific characteristics of the patient's condition. Continued monitoring and follow-up are also necessary in evaluating prognosis.
